# The efficacy of dexamethasone on pain and recovery after total hip arthroplasty: A systematic review and meta-analysis of randomized controlled trials

**DOI:** 10.1097/MD.0000000000010100

**Published:** 2018-03-30

**Authors:** Zheng-rui Fan, Jianxiong Ma, Xin-long Ma, Ying Wang, Lei Sun, Yan Wang, Ben-chao Dong

**Affiliations:** a Biomechanics Labs of Orthopaedics Institute, Tianjin Hospital; b Tianjin Hospital, Tianjin University; c Department of Orthopedics, Tianjin Medical University General Hospital, Tianjin, People's Republic of China.

**Keywords:** dexamethasone, meta-analysis, nausea, total hip arthroplasty, VAS score

## Abstract

**Background:**

Total hip arthroplasty (THA) perioperative dexamethasone treatment is still a controversial subject. We write this systematic review and meta-analysis to evaluate the efficacy of dexamethasone on pain and recovery after THA.

**Methods:**

Two researchers searched the relevant studies from Pubmed, Cochrane, and Embase. The research was reported according to the preferred reporting items for systematic reviews and meta-analysis (PRISMA) guidelines. Randomized controlled trials (RCTs) were included in our meta-analysis. At the same time, the assessment of the risk of bias was conducted according to the Cochrane Handbook for Systematic Reviews of Interventions version. The pooled data are processed by software RevMan 5.3.

**Result:**

In accordance with inclusion and exclusion, 3 studies with 207 patients were eligible and accepted into this meta-analysis. For RCTs, the risk of bias was evaluated by Cochrane Collaboration tool. Only one study did not have detection bias. Our study demonstrated that the dexamethasone group was more effective than the placebo group in term of visual analogue scale (VAS) score at 24 hours (*P* < .001), 48 hours (*P* = .04); opioid consumption (*P* < .001); length of stay (LOS, *P* < .001); and postoperative nausea (*P* = .001).

**Conclusion:**

Dexamethasone not only reduces postoperative pain scores and postoperative opioids consumption within 48 hours, but also reduces postoperative vomiting and effectively reduces LOS. However, we still need large sample size and high quality studies to explore the relationship between complications and dose response to give the final conclusion.

## 1 Introduction

For those patients who have hip joints disease, total hip arthroplasty (THA) is considered to be one of the most successful treatment options for end-stage osteoarthritis and other hip diseases.^[1]^ Generally, the THA will benefit rapid recovery, accelerate functional recovery, and improve patient satisfaction.^[2,3]^ Even so, most patients still suffer from moderate to severe pain after THA.^[4]^ Moreover, nausea and vomiting as a common sequelae of anesthesia seriously affect the life quality of patients after surgery.^[5]^ Since postoperative pain, nausea, and vomiting can lead to patients’ discomfort, reduced surgical satisfaction and extended length of stay (LOS),^[6]^ the management of adverse events will be largely beneficial for patients’ physiological function and mental recovery. Steroids and glucocorticoids can effectively relieve pain by controlling the inflammation of the wound site.^[7]^ Dexamethasone is a highly potent and long-term glucocorticoid which is widely used in the field of orthopedics. It can relieve pain by inhibiting peripheral phospholipase.^[5]^ Preoperative small dose of dexamethasone can not only reduce postoperative pain, but also effectively relieve the occurrence of nausea and vomiting.^[3,8]^ Because of its potential side effects, the application of preoperative glucocorticoids is still very controversial.^[9]^ Salerno and Hermann^[10]^ already demonstrated that dexamethasone was safe and effective in controlling pain. McKee^[11]^ believes that the effect of short-term dexamethasone treatment on femoral head necrosis is extremely small. On the other hand, some studies have reported the potential side effects of dexamethasone, such as injured sleep quality, increased risk of infection and early postoperative blood glucose elevation.^[12]^ So far, whether dexamethasone can reduce pain, relieve nausea and vomiting effectively, or is not still controversial. Therefore, we carried out this systemic review and meta-analysis to evaluate the safety and efficiency of dexamethasone in THA.

## 2 Methods

Our research was reported according to the preferred reporting items for systematic reviews and meta-analysis (PRISMA) guidelines. The study was approved by the ethics committee of the Tianjin Hospital of Tianjin.

### 2.1 Search strategy

**Figure 1:**
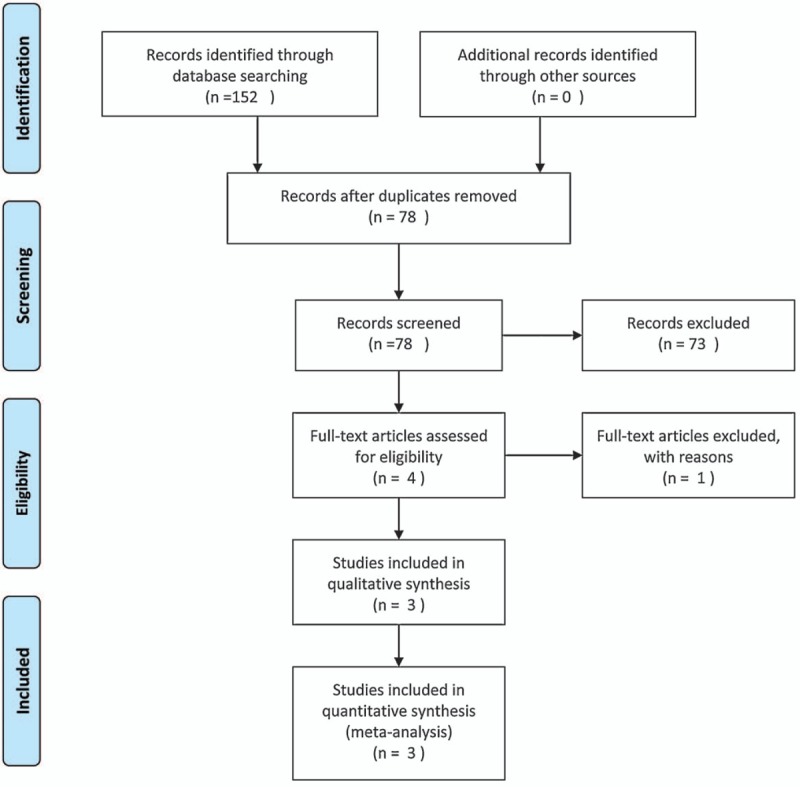
Search results and the selection procedure.

We searched 3 electronic databases (PubMed, EMBASE, and Cochrane Central Register of Controlled Trials) from 1998 to June 2017. A structured search was performed using the following search string: “dexamethasone” OR “hexadecadrol” AND (“THA” OR “THR” OR “total hip arthroplasty” OR “total hip replacement” OR “Arthroplasty, Replacement, Hip[Mesh]”) “Search key words included “dexamethasone,” “total hip arthroplasty,” “THA,” “total hip replacement,” and “pain control.” Each keyword is connected via a Boolean operator “AND” or “OR.” The retrieval process is presented in Fig. 1. No language and publication restrictions were applied.

### 2.2 Inclusion and exclusion criteria

Research can be incorporated if the following criteria are met: randomized controlled trials (RCT); the patient treated by THA; the experimental group received preoperative dexamethasone injection and control group received placebo or nothing; evaluation criteria include visual analogue scale (VAS) scores, opioid consumption, LOS, and postoperative nausea risk; and incomplete data, case reports, meeting summaries, or review articles should be excluded.

### 2.3 Literature selection

All searched studies were imported into endnote X7 and duplicate documents were deleted. And then, according to the title and abstract of the literature, irrelevant literatures were excluded. At last, 2 researchers removed the literature that meets the exclusion criteria. When the opinion is inconsistent, we discussed the decision with the senior reviewer.

### 2.4 Date extraction

Researchers independently distill the relevant items.

The data include first author names, published year, sample size, anesthesia types, and follow-up period. The main results of the data collection are as follows: pain, opioid consumption, hospital LOS, and PONV.

### 2.5 Assessment of study quality

Two investigators independently evaluated the literature-related risk of bias according to the Cochrane Handbook for Systematic Reviews of Interventions version. The evaluation criteria include the following 7: sequence generation, allocation concealment, blinding of participants, blinding of outcome assessor, incomplete outcome data, reporting bias, and other bias. If there is a discrepancy between the evaluations, a 3rd reviewer should be asked to join the discussion.

### 2.6 Data analysis

All data are processed by software RevMan 5.3. Variables are mainly divided into 2 types: dichotomous data and continuous data. The nausea was mainly expressed as dichotomous data. The odds ratio indicates the effect of intervention. The continuous various outcome was evaluated by mean difference (MD) or standard mean difference (SMD) with a 95% confidence interval (CI), like VAS scores, opioid consumption, and LOS. The chi-squared test, the value of *P*, and *I*^2^ tests were used to assess statistical heterogeneity. If *I*^2^ > 50%, *P* < .1, statistics was considered to be heterogeneous. When *P* ≥ .1 and *I*^2^ ≤ 50%, the fixed-effect model was performed for meta-analysis; otherwise, the random-effect model was used.

### 2.7 Subgroup analyses

Q and chi-squared tests in accordance with the values of *P* and *I*^2^ represent the heterogeneity of the included studies. The fix-effects model was used, if the *I*^2^ < 50% and *P* > .1. In contrast, we use a random-effects model to the meta-analysis. Subgroup analysis was used to explore the origin of heterogeneity.

## 3 Results

### 3.1 Search result

We searched 152 studies from Pubmed, Cochrane, and Embase, and then imported them into Endnote X7. No additional record was found when searching for references manually. A total of 78 studies were remained after removal of duplicates. According to the titles and abstracts, 73 studies were excluded. The last 4 studies were evaluated by reading the full texts, and one study was removed for failing to satisfy the related information. Finally, 3 RCTs^[12–14]^ were selected in meta- analysis. The PRISMA flow diagram is listed in Fig. 1.

### 3.2 Description of included studies

Some of the basic information of the studies was included in Table 1. A total of 207 samples were included; 110 patients were in the dexamethasone group; and 107 were in the control group. Intravenous dexamethasone was received in experimental group, while placebo was received in control group. All patients underwent unilateral hip arthroplasty and used patient control analgesia with opioid to treat pain. The follow-up of one study was unknown.

**Table 1:**
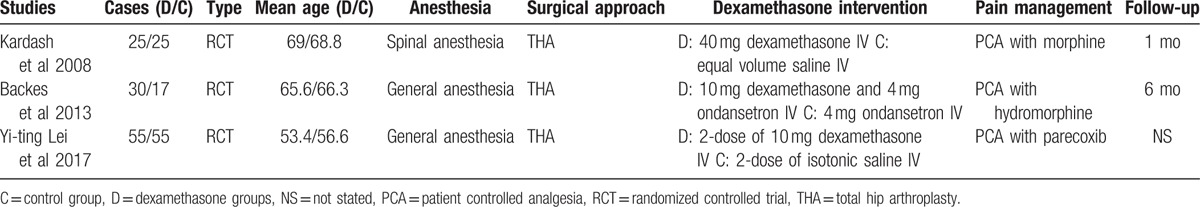
Description of included studies.

### 3.3 Risk of bias assessment

**Figure 2:**
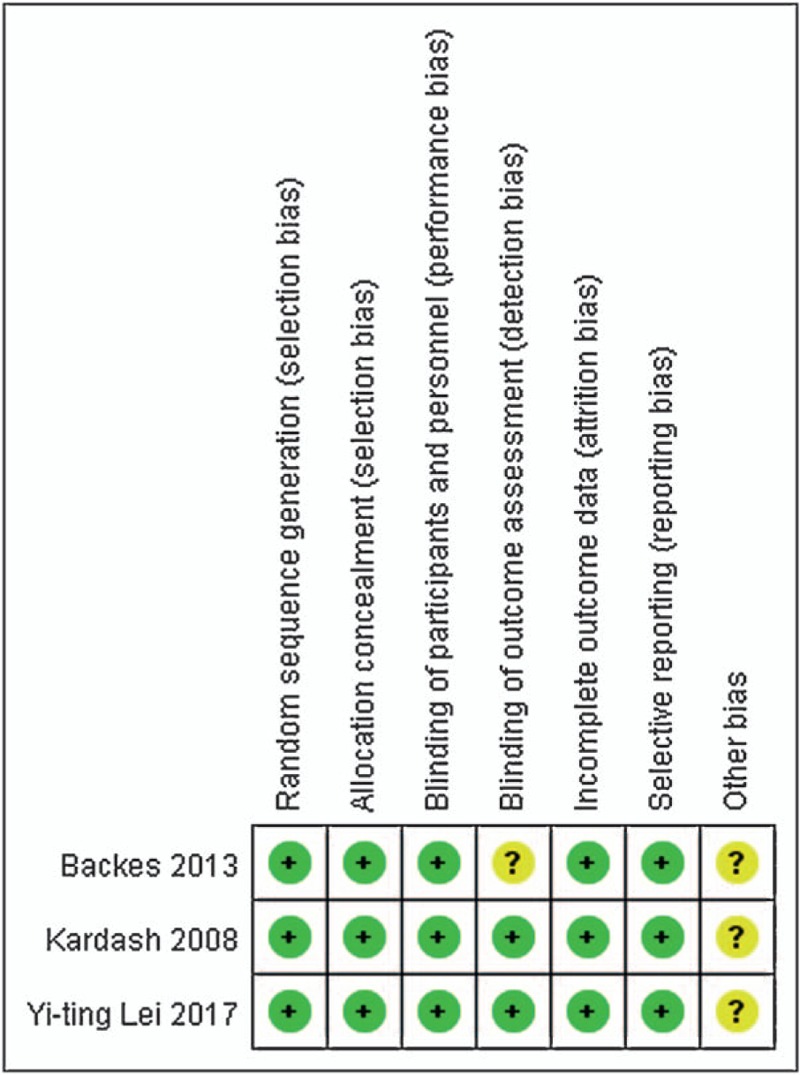
Methodological quality of the randomized controlled trials.

**Figure 3:**
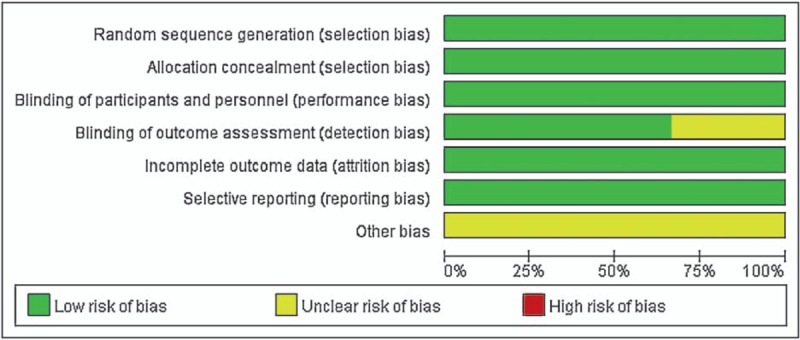
Risk of bias.

The RCTs^[12–14]^ were assessed by Cochrane Collaboration tool (Fig. 2). All RCTs provided explicit inclusion and exclusion criteria and suggest a randomized approach that the randomization algorithm is generated by a computer program. All studies are realized allocation concealment. All RCTs provided blinding of participants and personnel. In all studies, the risk of each biased project is a percentage. Percentage represents the degree of risk of bias for each item to varying degrees (Fig. 3).

### 3.4 Primary outcomes

#### 3.4.1 VAS score at 24 hours

**Figure 4:**
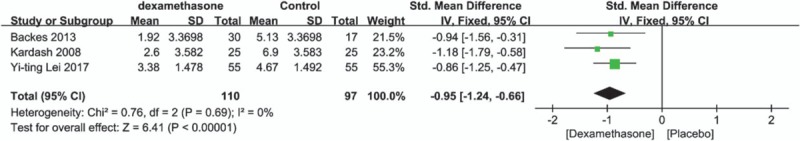
VAS score at 24 h after THA. THA = total hip arthroplasty, VAS = visual analogue scale.

Three studies^[12–14]^ assessed 24 hours VAS scores after THA. Because of no obvious heterogeneity between the studies (x^2^ = 0.76, df = 2, *I*^2^ = 0%, *P* = .69), the fixed-effects model was selected for using. Data summary analysis shows that VAS scores at 24 hours in experimental group were significantly lower than which in control group (SMD = −0.95, 95% CI: −1.24 to −0.66, *P* < .001; Fig. 4).

#### 3.4.2 VAS score at 48 hours

**Figure 5:**
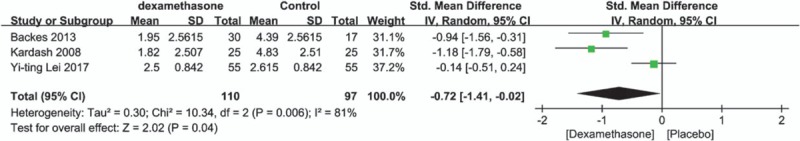
VAS score at 48 h after THA. THA = total hip arthroplasty, VAS = visual analogue scale.

The outcome of VAS scores at 48 hours after THA was provided by all 3 studies.^[12–14]^ Heterogeneity significantly exists between studies, so a random-effects model was applied (x^2^ = 10.34, df = 2, *I*^2^ = 81%, *P* = .006). The results of the analysis show that VAS scores at 48 hours in control group were significant higher than in dexamethasone group (SMD = −0.72, 95%CI: −1.41 to −0.02, *P* = .04; Fig. 5).

#### 3.4.3 Opioid consumption at 48 hours

**Figure 6:**
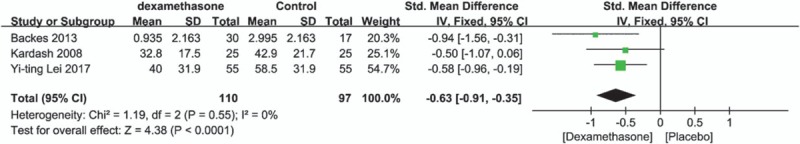
Opioid consumption at 48 h after THA. THA = total hip arthroplasty.

The opioid consumption at 48 hours was collected from 3 studies.^[12–14]^ There was no significant heterogeneity between 3 studies (x^2^ = 1.19, df = 2, *I*^2^ = 0%, *P* = .55); and then, the fixed-effect mode was used to count the data. The outcome of analysis demonstrated that opioid consumption at 48 hours in dexamethasone group was significantly lower than that in control group (SMD = −0.63, 95%CI: −0.91 to −0. 35, *P* < .001; Fig. 6).

#### 3.4.4 Length of hospital stay (LOS)

**Figure 7:**
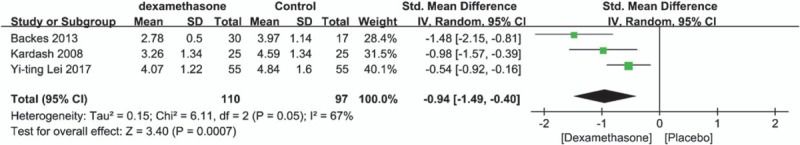
Length of hospital stay after THA. THA = total hip arthroplasty.

The length of hospital stay was provided by 3 studies.^[12–14]^ We found that the pooled results were heterogeneous (x^2^ = 6.11 df = 2, *I*^2^ = 67%, *P* = .05). Thence, a random-effects model was applied to count data. The outcome of analysis demonstrated that the length of hospital stay in dexamethasone group was significantly lower than that in control group (SMD = −0.94, 95%CI: −1.49 to −0.40, *P* < .001; Fig. 7).

#### 3.4.5 The incidence of nausea

**Figure 8:**
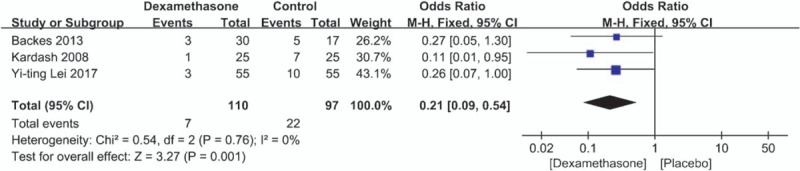
The incidence of nausea after THA. THA = total hip arthroplasty.

All 3 studies^[12–14]^ recorded the incidence of nausea. There was no significant heterogeneity between 3 studies (x^2^ = 0.54, df = 2, *I*^2^ = 0%, *P* = .76). Therefore, the fixed-effects model was applied to count data. The results of the analysis show that the incidence of nausea in control group was significant higher than in dexamethasone group (OR = 0.21, 95%CI: 0.09 to −0.54, *P* = .001; Fig. 8).

## 4 Discussion

At present, the incidence of arthritis is increasing as the aging of the population. THA as a treatment for hip osteoarthritis has been widely used. However, postoperative pain is still a tough trouble plaguing most patients. Several simple and straightforward interventions to manage each patient's predictable physiologic response to surgery make patients from the sick model to the well model and achieve rapid recovery joint arthroplasty. Dexamethasone, as a kind of antiemetics and potential analgesics, is such a simple and straightforward intervention to reduce PONV and relief pain to make arthroplasty patients from sick to well. However, there is still controversy whether dexamethasone contributes to postoperative recovery. Therefore, it is necessary to rely on evidence-based study to help the surgeon make correct clinical decisions.

This is a systematic review and meta-analysis of the effect of perioperative dexamethasone therapy on THA. Our pooled data analysis shows that dexamethasone was superior to control group in terms of VAS score, opioid consumption, LOS, and postoperative nausea.

VAS score is the primary assessment of our meta-analysis. The pooled data suggest that dexamethasone has a significant effect in relieving THA postoperative pain. At the same time, the risk of bias and high heterogeneity should be taken into account when interpreting the results. The current study shows that dexamethasone cannot effectively reduce postoperative pain, nausea, and vomiting. Christensen et al^[15]^ showed that dexamethasone did not effectively relieve the pain after TJA. However, dexamethasone as a long-acting glucocorticoid has been widely used in postoperative pain. Kim et al^[16]^ showed that injection of dexamethasone in brachial plexus can significantly reduce early postoperative pain, which is consistent with our statistical results. In our meta-analysis, dexamethasone can significantly reduce the postoperative pain score at the first 48 hours after THA. Backes et al^[12]^ demonstrated that dexamethasone group was more effective in reducing THA postoperative pain than the placebo group. Therefore, compared with the placebo group, dexamethasone group can provide more effective analgesia for patients after THA.

The total number of opioid consumption is also an important indicator of THA postoperative analgesic effect evaluation. In the first 48 hours after total joint replacement, 60% patients had a severe pain, and moderate pain accounted for 30%.^[17]^ Although a variety of analgesic methods are currently applied to postoperative pain management, most of them are insufficient in most cases. Additional opioids are used in pain management. Lei ^[8]^ reported that the opioid consumption in the dexamethasone group was significantly less than that in the placebo group. Our meta-analysis results are consistent with the results mentioned above. Our meta-analysis demonstrated that the consumption of opioids in the control group was significantly higher than that in the dexamethasone group after THA. Therefore, based on these results, we can draw conclusions clearly.

Our study showed that the incidence of nausea in control group was significant higher than in dexamethasone group. Lunn and Kehlet^[18]^ reported that steroids can reduce the incidence of PONV by central antiemetic effect. Liu et al^[19]^ believed that at least 5 mg dexamethasone can effectively reduce preoperative nausea and vomiting. Receiving THA can assist in injection of dexamethasone for analgesia. We also found that the risk of postoperative complications was reduced in part due to reduced opioid consumption and the use of dexamethasone. However, most physicians are still concerned about the side effects of dexamethasone and set a limitation in the use of dexamethasone in surgery. Studies^[20]^ have shown that there is no reliable evidence for the side effects of glucocorticoid after single dose administration. However, due to the small sample size of the 3 studies, no specific dose of dexamethasone was studied. Meanwhile, it is reported that early exercise can help postoperative functional recovery, reduce postoperative complications, and LOS.^[21]^ Lei et al^[14]^ demonstrated that the LOS in the dexamethasone group was significantly less than that in the placebo group and the data had statistical significance. Our meta-analysis is also consistent with its conclusions. There was a clear difference between the 2 groups. Our systematic review and meta-analysis still has some limitations:

Only 3 RCTs were included in the meta-analysis, the amount of sample data is relatively small; if there are more RCTs in the later stages, the effectiveness of our data will be improved.

As a result of THA postoperative recovery criteria, functional recovery results are important parameters. Due to lack of postoperative functional recovery data, we cannot conduct a meta-analysis about it.

All studies were followed for a short follow-up study and should be followed for a long-term follow-up study.

The publication bias in the current meta-analysis may affect the outcome.

Because of the heterogeneity of VAS at 48 hours (*I*^2^ = 81%), only 3 studies reported VAS at 48 hours, so subgroup analysis cannot be applied.

The sensitivity analysis was conducted to search the source of heterogeneity. If the study by Lei et al^[14]^ of VAS score at 48 hours (*I*^2^ = 81%) was excluded, the heterogeneity of VAS score at 48 hours (95%CI, −1.50 to −0.63; *I*^2^ = 0%) will become significant. So, we believe that the source of heterogeneity was from this study. In the study of Lei et al,^[14]^ the patients received 2-dose of 10 mg IV-dexamethasone, the 1st-dose was injected after the general anesthesia; the 2nd was administrated when patients returned to the ward. So, the heterogeneity may emerge from the moment of injection of dexamethasone. We applied the preferred reporting items for systematic reviews and meta-analyses (PRISMA) guidelines and Cochrane Handbook to assess the quality of the results published in all included studies to ensure that the results of our meta-analysis were reliable and veritable. Despite the above limitations, our study is the most recent RCT of meta-analysis to evaluate the first efficiency and the safety of dexamethasone in THA. There is also a need for a large number of RCTs to be verified.

## 5 Conclusion

Dexamethasone not only reduces postoperative pain scores and postoperative opioids consumption within 48 hours, but also reduces postoperative vomiting and effectively reduces LOS. However, we still need large sample size and high quality studies to explore the relationship between complications and dose response to give the final conclusion.

## Author contributions

**Conceptualization:** Y. Wang, Z-R. Fan.

**Formal analysis:** J. Ma.

**Funding acquisition:** X-L. Ma.

**Investigation:** X-L. Ma.

**Methodology:** Y. Wang.

**Project administration:** X-L. Ma.

**Resources:** B-C. Dong.

**Resources:** Y. Wang.

**Validation:** L. Sun.

**Writing – original draft:** Z-R. Fan.

**Writing – review & editing:** Z-R. Fan.
